# Intraspecific Variation in Wood Anatomical, Hydraulic, and Foliar Traits in Ten European Beech Provenances Differing in Growth Yield

**DOI:** 10.3389/fpls.2016.00791

**Published:** 2016-06-15

**Authors:** Peter Hajek, Daniel Kurjak, Georg von Wühlisch, Sylvain Delzon, Bernhard Schuldt

**Affiliations:** ^1^Plant Ecology, Albrecht von Haller Institute for Plant Sciences, University of GöttingenGöttingen, Germany; ^2^Faculty of Forestry, Technical University in ZvolenZvolen, Slovakia; ^3^Federal Research Institute for Rural Areas, Forestry and Fisheries, Thuenen Institute for Forest GeneticsGroßhansdorf, Germany; ^4^UMR BIOGECO Institut National de la Recherche Agronomique-UB, University of BordeauxTalence, France

**Keywords:** adaptive capacity, *Fagus sylvatica* L., genetic variability, hydraulic conductivity, leaf morphology, phenotypic plasticity, provenance trial, vulnerability to cavitation

## Abstract

In angiosperms, many studies have described the inter-specific variability of hydraulic-related traits and little is known at the intra-specific level. This information is however mandatory to assess the adaptive capacities of tree populations in the context of increasing drought frequency and severity. Ten 20-year old European beech (*Fagus sylvatica* L.) provenances representing the entire distribution range throughout Europe and differing significantly in aboveground biomass increment (ABI) by a factor of up to four were investigated for branch wood anatomical, hydraulic, and foliar traits in a provenance trial located in Northern Europe. We quantified to which extend xylem hydraulic and leaf traits are under genetic control and tested whether the xylem hydraulic properties (hydraulic efficiency and safety) trades off with yield and wood anatomical and leaf traits. Our results showed that only three out of 22 investigated ecophysiological traits showed significant genetic differentiations between provenances, namely vessel density (VD), the xylem pressure causing 88% loss of hydraulic conductance and mean leaf size. Depending of the ecophysiological traits measured, genetic differentiation between populations explained 0–14% of total phenotypic variation, while intra-population variability was higher than inter-population variability. Most wood anatomical traits and some foliar traits were additionally related to the climate of provenance origin. The lumen to sapwood area ratio, vessel diameter, theoretical specific conductivity and theoretical leaf-specific conductivity as well as the C:N-ratio increased with climatic aridity at the place of origin while the carbon isotope signature (δ^13^C) decreased. Contrary to our assumption, none of the wood anatomical traits were related to embolism resistance but were strong determinants of hydraulic efficiency. Although ABI was associated with both VD and δ^13^C, both hydraulic efficiency and embolism resistance were unrelated, disproving the assumed trade-off between hydraulic efficiency and safety. European beech seems to compensate increasing water stress with growing size mainly by adjusting vessel number and not vessel diameter. In conclusion, European beech has a high potential capacity to cope with climate change due to the high degree of intra-population genetic variability.

## Introduction

European beech (*Fagus sylvatica* L.) dominated the natural vegetation types of forests in Central Europe for centuries, forming large stands of resilient forest ecosystems (Ellenberg and Leuschner, 2010). Despite the competitive superiority of European beech for tree populations in temperate forests, this species is more vulnerable to drought-induced stem growth reductions than other temperate broad-leaved trees (Leuschner et al., 2001; Zimmermann et al., 2015). Consequently, the Central European beech populations are expected to suffer high mortality rates probably altering the distribution range at its dry distributional limit as a consequence of increased physiological stress due to a higher risk of drought exposure associated with recent climate change.

According to its large geographic distributional range, European beech is expected to exhibit substantial genetic diversity (Bolte et al., 2007). The inherent capability of this species to survive and reproduce successfully across such a wide range of habitats is either maintained by long-term adaptation (i.e., genotypic variation) or short-term acclimation (i.e., phenotypic plasticity; Lindner et al., 2010; Kremer et al., 2014). Consequently, evolutionary adaptation may have caused the selection of ecotypes adapted to the regional climatic conditions, which is manifested in phenotypic variation of plant functional traits across various provenances (Hamrick, 2004). Despite an increasing amount of studies on functional traits which are known to be related to drought resistance in beech, e.g., leaf stomatal features (Stojnić et al., 2015), wood structure (Eilmann et al., 2014), resistance to xylem cavitation (Herbette et al., 2010, Wortemann et al., 2011), or hydraulic architecture (Aranda et al., 2015; Schuldt et al., 2016), still very little information is available on the relevance of phenotypic plasticity or genetic variation of traits on the adaptation of populations.

The high trait plasticity and remarkable regeneration potential of beech trees after disturbance leads to the assumption that beech is able to bear water shortage to a certain degree (Kahle, 2006; van der Werf et al., 2007). However, other studies report on drought susceptibility or dramatic yield loss and regeneration periods of decades subsequent to drought events and there is still an ongoing debate on the response of beech populations to drought exposure (Leuschner et al., 2001; Peuke et al., 2002; Leuzinger et al., 2005; Bréda et al., 2006; Jump et al., 2006). The mechanisms behind the adaptation and adaptability of beech to drought is a main challenge for a better understanding of the effects of climate change on this economically and ecologically important tree species. Because productivity is closely coupled with hydraulic efficiency (Hajek et al., 2014; Hoeber et al., 2014; Kotowska et al., 2015) and may only be achieved at the cost of hydraulic safety (Cochard et al., 2007), genetic differentiation in wood anatomical and hydraulic traits should be most pronounced between provenances differing in yield. However, the way how different provenances of the same tree species cope with such trade-offs still remains poorly investigated.

The main objective of this study was to investigate the genetic differentiation in vulnerability to xylem embolism and other related hydraulic and foliar properties across ten European beech provenances differing in growth performance. We further aimed to investigate the relationships and potential trade-offs between hydraulic, wood anatomical and leaf traits within species. We hypothesized that (i) drought-related traits are under genetic control and therefore populations are locally adapted to their environment, (ii) hydraulic efficiency and embolism resistance are related to anatomical traits, and (iii) a high hydraulic efficiency leads to high growth rates at the cost of xylem safety.

## Materials and methods

### Experimental site, plant material, and microclimatic conditions at the place of origin

The field trial studied is part of the International Beech Provenance Trial Series 1993/1995 under EU funding (AIR3-CT94-2091), which investigates the role of the genetic variation of beech for adaptability, productivity and selected ecosystem functions considering risks of global climate change (von Wuehlisch et al., 1998). For the present study conducted in August 2014, the common-garden field trial with 100 different European beech (*Fagus sylvatica* L.) provenances established in 1995 in Northern Germany (Schleswig-Holstein) with 2-year old saplings near the Schädtbek Experimental Farm (54° 18′N, 10°16′E, 40 m a.s.l.) was used. The climate at the site is oceanic, moderately cold with a mean annual temperature (MAT) of 8.3°C, a mean annual precipitation (MAP) of 742 mm and a mean early growing season precipitation from April to June (MSP) of 149 mm (data obtained from the German Meteorological Service). The common garden trial consists of 10 × 10 m plots planted with 50 beech saplings per provenance in a 3-times replicated randomized block design. The beech trees are arranged in a rectangular grid with a planting distance of 2 m between and 1 m within rows. The trial is surrounded by a single bordering tree row serving as buffer zone to avoid edge effects. As all provenances were grown in a single environment (common garden), we were able to assess the genetic differentiations between provenances for several functional traits. The common garden experienced no management operations since plantation establishment in 1995, resulting in competitive selection among tree individuals leading to heterogeneity in plot characteristics.

From the 100 available provenances, ten provenances differing in aboveground growth increment (Figure S1) were selected in order to cover not only a gradient in growth yield but also a climatic gradient at the place of origin (Figure S2). By this selection, we covered provenances from different geographic regions and climates throughout Europe with a broad range of MAT (3.4–15.3°C) and MAP (575–1080 mm; Table 1). Mean annual climate data from 1950 to 2000 at the place of origin for each of the ten provenances were obtained from the WorldClim database with 30 arc-seconds resolution (Hijmans et al., 2005). In order to estimate climate dryness at the place of origin, we used the WorldClim database to access the global aridity index (GAI, Zomer et al., 2008) and calculated the forest aridity index (FAI) according to Fuehrer et al. (2011) as FAI = 100 × *T*_Jul−Aug_/(*P*_Mai−Jul_ + *P*_Jul−Aug_), where *T* is the temperature and *P* the precipitation of the associated interval. Since the atmospheric evaporative demand in the growing season is highest in mid-summer (July and August), the July precipitation was weighted by a factor of two in the denominator. We further calculated Ellenberg's climate quotient (EQ, Ellenberg and Leuschner, 2010) as EQ = (*T*_Jul_/*P*_annual_) × 1000. All measures of water availability and climatic aridity at the places of provenance origin were highly interrelated (data not shown). We therefore decided to use the forest aridity index (FAI) as integrate variable for all subsequent analyses.

**Table 1:** **Climatic data at the place of origin of the ten *Fagus sylvatica* provenances including elevation, mean annual temperature (MAT), mean annual precipitation (MAP), mean early growing season precipitation from April to June (MSP), Ellenberg's climate quotient (EQ), the global aridity index (GAI) and the forest aridity index (FAI)**.

Acronym	Country, location	Coordinates	Elevation	MAT	MAP	MSP	EQ	GAI	FAI
			m	°C	mm yr^−1^	mm yr^−1^			
BG	Bulgaria, Ribaritza, Lovetch	42°55′ N, 24°16′ E	900	15.3	945	214	32.12	0.67	6.09
CZ	Czech Republic, Kladanska, Lazne Kynzvart	50°2′ N, 12°37′ E	690	6.0	750	218	16.30	1.38	3.03
DE-BB	Germany, Gransee, Abt. 3082 a1	53°0′ N, 13°10′ E	70	8.5	575	162	31.23	0.81	5.75
DE-SH	Germany, Schleswig Holstein, Lensahn	54°12′ N, 10°45′ E	80	8.3	700	162	22.41	1.16	4.63
ES	Spain, Anguiano La Rioja	42°15′ N, 2°45′ W	950	9.9	860	170	33.79	0.66	9.16
RO	Romania, Beliu-Arad /Groseni	46°29′ N, 22°9′ E	575	9.5	820	222	30.82	0.73	5.38
SE	Sweden, Ryssberget, Sölvesborg	56°5′ N, 14°36′ E	90	8.0	750	125	26.63	1.10	5.67
SK	Slovakia, Trencin	48°53′ N, 18°0′ E	200	9.0	670	211	27.99	0.79	4.59
SL	Slovenia, Rogaska Slatina	46°18′ N, 15°36′ E	420	9.0	1050	286	19.67	1.37	3.68
UA	Ukraine, Svaljava Polana	48°38′ N, 19°30′ E	1150	3.4	1080	354	11.53	1.76	2.00

*Mean annual climate data from 1950 to 2000 were obtained from the WorldClim database with 30 arc-seconds resolution (Hijmans et al., 2005)*.

Provenances were selected according to differences in aboveground growth increment based on a tree inventory in January 2013 (Figure S1). Within a given provenance, ten tree individuals of comparable size close to the population average (diameter at breast height and tree height) were selected assuming uniform growth conditions (site conditions and intraspecific competition) and consequently representing the average growth increment of a given provenance, yielding 100 processed tree individuals in total. A 2- to 4-year-old branch segment (mean ± SE : 2.25 ± 0.05 year) was collected in August 2014 from the uppermost canopy of each selected tree individual with a long-reaching telescope pruner and recut to approximately 50 cm length on the ground. Selected segments were defoliated and immediately transferred to plastic tubes containing deionized water and Micropur (Katadyn, Wallisellen, Switzerland) to prevent microbial activity and stored at 4°C until further processing within 4 weeks. All leaves of the respective segments were stored separately for foliar analyses. A list of all measured traits, their symbols and units are given in Table 2.

**Table 2:** **List of variables with acronyms and units employed**.

Variable	Unit	Definition
DBH	cm	Diameter at breast height
Height	m	Tree height
AGB	kg	Aboveground biomass
BAI	cm^2^ yr^−1^	Basal area increment
ABI	kg yr^−1^	Aboveground biomass increment
*P*_12_	MPa	Xylem pressure at 12% loss of hydraulic conductance
*P*_50_	MPa	Xylem pressure at 50% loss of hydraulic conductance
*P*_88_	MPa	Xylem pressure at 88% loss of hydraulic conductance
*A*_xylem_	mm^2^	Branch sapwood area
$K_{\text{S}}^{\text{emp}}$	kg m^−1^ MPa^−1^ s^−1^	Empirical specific conductivity
$K_{\text{S}}^{\text{theo}}$	kg m^−1^ MPa^−1^ s^−1^	Theoretical specific conductivity
$K_{\text{L}}^{\text{emp}}$		Empirical leaf-specific conductivity
$K_{\text{L}}^{\text{theo}}$	10^−4^ kg m^−1^ MPa^−1^ s^−1^	Theoretical leaf-specific conductivity
BA	yr	Branch age
*A*_growth_	mm^2^ yr^−1^	Annual branch sapwood area increment
*A*_lumen_ : *A*_xylem_	%	Lumen to sapwood area ratio
VD	n mm^−2^	Vessel density
*D*	μm	Vessel diameter
*D*_h_	μm	Hydraulically weighted vessel diameter
*A*_leaf_	cm^2^	Mean leaf size
SLA	cm^2^ g^−1^	Specific leaf area
*A*_S_ : *A*_L_	10^−4^ m^2^ m^−2^	Sapwood to leaf area ratio (Huber value)
C:N		Carbon to nitrogen ratio
δ^13^C	‰	Carbon isotope signature
Ca_mass_	g kg^−1^	Mass-specific foliar calcium content
K_mass_	g kg^−1^	Mass-specific foliar potassium content
Mg_mass_	g kg^−1^	Mass-specific foliar magnesium content
N_mass_	g kg^−1^	Mass-specific foliar nitrogen content
P_mass_	g kg^−1^	Mass-specific foliar phospor content

### Timber volume, above-ground biomass, and basal area increment

Aboveground growth performance (i.e., stem increment and height growth) of the respective genotypes under the local environment was evaluated from diameter at breast height (DBH, cm) and tree height (m). Aboveground productivity expressed as aboveground biomass increment (ABI, kg yr^−1^) was calculated for the entire growth period (1995–2014), and basal area increment (BAI, cm^2^ yr^−1^) from two inventories in January 2013 and August 2014, respectively. For calculating the volume of stems and branches of at least 7 cm in diameter (standing volume of timber), we used the allometric equation *V* = π × ((*D*/100)/2)^2^ × *H* × *f*, where *V* is the volume of timber (m^3^), *D* the diameter at breast height (cm), *H* tree height (m), and *f* an empirically derived form factor for beech trees (Bergel, 1973) with *f* = 0.4039 + (0.0017335 × *H*) + (1.1267/*H*)–(118.188/*D*^3^) + (4.2 × 10^6^ × *D*^2^). Above-ground biomass was estimated from an empirical equation given by Wutzler et al. (2008) as AGB = 0.00523 × *D*^2.12^ × *H*^0.655^.

### Leaf-related measurements

From each branch segment, all leaves were removed from the basipetal segment upwards to determine mean leaf size (*A*_leaf_, cm^2^) and cumulative leaf area (*A*_L_, m^2^) using a flatbed scanner and the WinFOLIA software (Régent Instruments, Quebec, Canada). Per branch segment, 19 to 194 leaves were scanned, yielding 6008 leaves in total. Specific leaf area (SLA, cm^2^ g^−1^) was calculated by dividing the total leaf area by the leaf dry weight (70°C, 48 h). The Huber value, i.e., sapwood to leaf area ratio (*A*_S_ : *A*_L_, 10^−4^ m^2^ m^−2^), was calculated by dividing maximal sapwood area by *A*_L_. Subsequently, leaf samples were ground and the leaf dry mass analyzed for foliar *C* and *N* concentrations as well as the carbon isotope signature (δ^13^C, ‰) by using a Delta Plus Isotope mass ratio spectrometer (Finnigan MAT, Bremen, Germany), a Conflo III interface (Thermo Electron Corporation, Bremen, Germany) and a NA2500 elemental analyser (CE-Instruments, Rodano, Milano, Italy) using standard δ notion: δ = (*R*_sample_/*R*_standard_–1) × 1000 (‰) at the Centre for Stable Isotope Research and Analysis (KOSI), University of Göttingen. The foliar concentrations of Ca, K, Mg, and P were measured by ICP analysis (Optima 5300 DV, PerkinElmer Inc., USA).

### Branch xylem anatomy, theoretical conductivity, and growth rate

Measurements of anatomical parameters were carried out on 9–10 branch segments from the basipetal end of the samples used for the hydraulic measurements of each provenance, yielding 98 samples in total. Prior to cutting semi-thin (10–20 μm) transverse sections with a sliding microtome (G.S.L.1, Schenkung Dapples, Zürich, Switzerland), the ethanol-stored (70%) segments were completely stained with a safranin solution (1 in 50% ethanol, Merck, Darmstadt, Germany) and subsequently embedded in Euparal medium. For digitalization, a stereo-microscope equipped with an automatic stage was used (SteREOV20, Carl Zeiss MicroImaging GmbH, Jena, Germany; Software: AxioVision v4.8.2, Carl Zeiss MicroImaging GmbH, Jena, Germany), enabling a time-efficient digitalization of the complete cross-section at 100x magnification. Image analysis was performed using the software Adobe Photoshop CS2 (Version 9.0, Adobe Systems Incorporated, USA) and ImageJ (v1.44p, http://rsb.info.nih.gov/ij) applying the particle analysis function. For all subsequent calculations, the complete xylem cross-section without pith and bark was analyzed, yielding 888,358 analyzed vessels in total. The following parameters were calculated: idealized vessel diameter (*D*, μm) as obtained from major (*a*) and minor (*b*) vessel radii according to Lewis and Boose (1995) as *D* = ((32 × (*a* × *b*)^3^)/(*a*^2^ + *b*^2^))^¼^, vessel density (VD, n mm^−2^) and cumulative vessel lumen area. The lumen to sapwood area ratio (*A*_lumen_ : *A*_xylem_, %), was obtained by dividing cumulative vessel lumen area by the corresponding sapwood area. The diameter of individual vessels was used to calculate hydraulically-weighted vessel diameter (*D*_h_, μm) according to Sperry et al. (1994) as *D*_h_ = Σ*D*^5^/Σ*D*^4^. Theoretical specific conductivity ($K_{\text{S}}^{\text{theo}}$, kg m^−1^ MPa^−1^ s^−1^) was calculated according to the Hagen-Poiseuille equation as $K_{\text{S}}^{\text{theo}}$ = (((π × Σ*D*^4^)/128 η) × ρ)/*A*_xylem_, where η is the viscosity of water (1.002 10^−9^ MPa s), ρ the density of water (998.2 kg m^−3^), both at 20°C, and *A*_xylem_ (m^2^) the corresponding xylem area without pith and bark. Branch growth rate (*A*_growth_, mm^2^ yr^−1^) was calculated by dividing *A*_xylem_ by the number of growth rings (i.e., branch age, BA). In addition to $K_{\text{S}}^{\text{theo}}$, theoretical leaf-specific conductivity was calculated by division by *A*_L_ as $K_{\text{L}}^{\text{theo}}$ = (((π × Σ*D*^4^)/128 η) × ρ)/*A*_L_.

### Hydraulic conductivity measurement

Hydraulic traits were measured in ten branch segments (mean diameter ± SE: 6.83 ± 0.06 mm) per provenance using the Xyl'em apparatus (Bronkhorst, Montigny-les-Cormeilles, France). In the laboratory, all lateral branches were cut off and the scares sealed with quick-drying superglue (Loctite 431, Henkel, Düsseldorf, Germany) applicable to wet surfaces. Subsequently, the segments were shortened to a length of 290.5 ± 0.8 mm (mean ± SE). For the determination of maximal hydraulic conductivity (*K*_h_, kg m MPa^−1^ s^−1^) at 6 kPa, demineralized filtered (0.22 μm) and degassed water (10 mM KCl and 1 mM CaCO_3_) was used, interrupted by three 10-min flushes at 120 kPa to assure removal off all potential emboli. The diameter of each segment was measured twice at the basipetal and distal end, and at four positions along the segment. The following regression coefficients were used to calculate sapwood area without pith and bark for a given beech branch segment diameter according to Schuldt et al. (2016): *A*_xylem_ = −3.715 + 0.770 *A*_cross_. Subsequently, empirical specific conductivity ($K_{\text{S}}^{\text{emp}}$, kg m^−1^ MPa^−1^ s^−1^) was calculated by dividing *K*_h_ by the maximal basipetal, and not average, sapwood area (Hajek et al., 2014; Hoeber et al., 2014; Schuldt et al., 2016). *K*_h_ was further used to calculate empirical leaf-specific conductivity ($K_{\text{L}}^{\text{emp}}$, kg m^−1^ MPa^−1^ s^−1^) by division by *A*_L_.

### Xylem resistance to cavitation

Vulnerability to xylem cavitation was determined on 8 to 10 branch samples (replicated trees) per provenance using the Cavitron technique (Cochard et al., 2005), yielding 94 samples in total. Segments with a standardized length of 27.5 cm were mounted in a custom-built honeycomb rotor chamber of the Cavitron, which uses a commercially available centrifuge as basis (Sorvall RC-5C, Thermo Fisher Scientific, Waltham, MA, USA), and spun at defined velocities recorded with the software CaviSoft (version 4.0.1.3, University of Bordeaux, France). Conductivity measurements started at 1.0 MPa and were stepwise repeated at intervals of 0.2 to 0.3 MPa until the percent loss of conductivity (PLC) reached at least 90%. All vulnerability curves of the present study measured on 27.5 cm long segments were s-shaped, indicating that no open vessels were present. For each branch segment, a sigmoid function (Willigen and Pammenter, 1998) was fitted to describe the relationship between PLC and xylem pressure using the expression PLC = 100/(1 + exp(*s*/25 × (*P*_i_ – *P*_50_))), where *P*_50_ (MPa) is the xylem tension causing 50% loss of hydraulic conductivity and *s* (% MPa^−1^) is the slope of the curve at the inflexion point. The xylem pressures causing 12% (*P*_12_) and 88% (*P*_88_) loss of conductivity were calculated as well.

### Statistical analysis

In order to assess the significance of differentiation between provenances for all functional traits, we used linear mixed effect models (LME) starting with a random effect model without any fixed effect but with “provenance” and “block” added as a random effects using the “lme” function of the R package “nlme” according to the following model [1]: *Y*_*ijk*_ = μ + *P*_*i*_ + *b*_*j*_ + ε_*ijk*_, where *Y*_*ijk*_ is the observation of tree individual *k* for one of the analyzed characters from provenance *i* and block *j*, μ is the overall mean, and *P*_i_ the error term for provenance, *b*_j_ for block and ε_ijk_ for the residual variation. During the analysis, normal distribution of the residuals and homogeneity of variance were assessed visually using residual diagnostics and quantile-quantile plots; some data ($K_{\text{L}}^{\text{emp}}$, $K_{\text{L}}^{\text{theo}}$, *A*_S_ : *A*_L_) had to be log-transformed in order to achieve normal distribution. In order to test whether the variance component accounting for genetic differentiation is significantly different from zero, a likelihood ratio test (LRT) was performed against a reduced model without a random effect for provenance using the restricted maximum likelihood (REML) method. As under those conditions the LRT is performed on the boundary of the parameter space, the resulting *P*-values had to be corrected by multiplying them by 0.5 (Verbeke and Molenberghs, 2009).

To quantify the influence of climate at the place of origin, FAI was added to the LME as fixed variable according to the following model [2]: *Y*_*ijk*_ = α + β FAI_*i*_ + *P*_*i*_ + *b*_*j*_ + ε_*ijk*_, where α is the intercept and β the slope of the linear model with FAI_*i*_, which all three together determine μ of model [1]. A significant influence of climate at provenance origin was determined by a LRT using the maximum likelihood (ML) method.

We further calculated coefficients of variation for within-provenance variation (CV_intra_) and between-provenance variation (CV_inter_) for each trait in order to allocate total measured trait variation to a genetic component (CV_inter_) and a predominantly phenotypic component (CV_intra_); the between-provenance variability (CV_inter_) was calculated from the between-provenance standard deviation (SD) and the overall mean value. Additionally, the ratio of provenance variance component to total variance was calculated using the R package “varComp” according to a variance component analyses with the program “lme” to calculate the proportion of total variance ($\sigma_{\text{total}}^{2}$) explained by the variability between provenances ($\sigma_{\text{inter}}^{2}$), replicated randomly distributed “blocks” ($\sigma_{\text{block}}^{2}$) and residual variance within provenances ($\sigma_{\text{intra}}^{2}$). Inter-population variance component (VC_inter_) was calculated according to VC_inter_ = ($\sigma_{\text{inter}}^{2}$/($\sigma_{\text{inter}}^{2}$ + $\sigma_{\text{block}}^{2}$ + $\sigma_{\text{intra}}^{2}$)) × 100, variance component between replicated plots as VC_block_ = ($\sigma_{\text{block}}^{2}$/($\sigma_{\text{inter}}^{2}$ + $\sigma_{\text{block}}^{2}$ + $\sigma_{\text{intra}}^{2}$)) × 100, and intra-population variance component (VC_intra_) as VC_intra_ = ($\sigma_{\text{intra}}^{2}$/($\sigma_{\text{intra}}^{2}$ + $\sigma_{\text{block}}^{2}$ + $\sigma_{\text{inter}}^{2}$)) × 100, all in percentage.

Pearson correlation analysis was used to test for inter-relationships between different branch traits of the trees and for detecting relationships between traits based on data pooled across all provenances. Applying correlations to raw measurements for such exploratory analysis may mix several sources of variation and result in inflated degrees of freedom and potentially overestimation of significance. Statistical analyses were performed with the software R (version 3.1.3, R Development Core Team, 2011), and all linear and non-linear regression analyses were carried out with the software Xact 8.03 (SciLab, Hamburg, Germany).

## Results

### Genetic differentiation between provenances

The estimate of aboveground growth potential as inferred from the basal area increment (BAI) from January 2013 to August 2014 and aboveground biomass increment (ABI) for the entire length of the experiment from August 1995 to August 2014 revealed large significant differences between the ten selected beech provenances (Table 3). The provenances with highest ABI from Slovakia and Ukraine (SK: 3.6; UA 4.0 kg yr^−1^, *P* < 0.001; Table S1) showed four times higher growth rates than the provenances with lowest growth rates from Slovenia and Sweden (SL: 1.02 and SE: 1.69 kg yr^−1^; Table S1). Both provenances had the highest radial growth rates (SK: 33.36 and UA: 23.20 cm^2^ yr^−1^) and grew more than seven-fold compared to the two provenances with lowest basal area increment (ES: 4.34 and SL: 5.68 cm^2^ yr^−1^; Table S1). The other six provenances reached intermediate growth rates (13.05–22.55 cm^2^ yr^−1^). ABI and BAI scaled highly positive with tree size (DBH, *r*^2^ = 0.98, *P* < 0.001 and *r*^2^ = 0.73, *P* < 0.001; height, *r*^2^ = 0.66, *P* < 0.01 and *r*^2^ = 0.31, *P* < 0.001; **Table 5**).

**Table 3:** **Results of a random effects model on the genetic differentiation between provenances and the coefficient of variation for all traits measured for the variability between provenances (CV_inter_) and within provenances (CV_intra_)**.

GENETIC DIFFERENTATION BETWEEN PROVENANCES								
Variable	CV_inter_	CV_intra_	VC_inter_	VC_block_	VC_intra_	Δ_i_	LR	*P*
**STAND CHARACTERISTICS**								
DBH	15.46	11.00	64.23	0.96	34.81	24.19	26.19	**<0.001**
Height	11.95	10.52	49.82	11.48	38.70	11.40	13.40	**<0.001**
AGB	36.98	25.44	66.73	0.00	33.27	32.88	34.88	**<0.001**
**GROWTH-RELATED TRAITS**								
BAI	54.20	56.81	40.51	0.36	59.13	10.42	12.42	**<0.001**
ABI	37.00	25.44	66.79	0.00	33.21	32.89	34.89	**<0.001**
**HYDRAULIC PROPERTIES**								
*P*_12_	7.88	14.51	8.38	25.20	66.43	1.41	0.59	0.22
*P*_50_	4.69	8.12	9.86	23.80	66.34	1.20	0.80	0.19
*P*_88_	4.87	7.20	8.38	25.20	66.43	2.18	4.18	**0.02**
$K_{\text{S}}^{\text{emp}}$	9.65	27.59	0.00	8.78	91.22	2.00	0.00	0.50
$K_{\text{S}}^{\text{theo}}$	15.69	35.52	6.93	0.00	93.07	0.57	1.43	0.12
$K_{\text{L}}^{\text{emp}}$	17.37	50.38	0.00	9.35	90.65	2.00	0.00	0.50
$K_{\text{L}}^{\text{theo}}$	21.45	65.24	0.00	4.99	95.01	2.00	0.00	0.50
**WOOD ANATOMY**								
BA	7.63	19.81	0.99	0.00	99.01	1.96	0.04	0.42
*A*_growth_	4.65	23.86	0.00	0.00	100.00	2.00	0.00	0.50
*A*_lumen_ : *A*_xylem_	9.45	19.93	9.57	0.00	90.43	−0.56	2.56	***0.05***
VD	6.72	12.58	14.02	0.00	85.98	2.67	4.67	**0.02**
*D*	4.09	8.32	10.10	0.00	89.90	−0.51	2.51	***0.06***
*D*_h_	3.75	9.14	5.31	0.00	94.69	1.20	0.80	0.19
**LEAF MORPHOLOGY**								
*A*_leaf_	9.07	18.08	12.09	0.00	87.91	0.77	2.77	**0.05**
SLA	7.28	23.88	0.00	3.86	96.14	2.00	0.00	0.50
*A*_S_ : *A*_L_	16.03	39.55	2.75	1.06	96.19	1.81	0.19	0.33
**LEAF CHEMISTRY**								
C:N	3.02	6.56	9.77	0.00	90.23	0.37	2.37	***0.06***
δ^13^C	1.16	2.73	6.48	0.00	93.52	0.88	1.12	0.14
Ca_mass_	5.67	21.72	0.00	0.00	100.00	2.00	0.00	0.50
K_mass_	11.43	23.55	9.85	0.00	90.15	0.20	2.20	***0.07***
Mg_mass_	9.12	24.36	1.66	0.00	98.34	1.90	0.10	0.38
P_mass_	3.81	13.12	0.00	0.00	100.00	2.00	0.00	0.50

*Given is the ratio of inter-population (VC_inter_) variance component between replicated plots within a given provenance (VC_plot_) and intra-population (VC_intra_) variance component to total variance, all in percentage. The results of a likelihood ratio test of a linear mixed effect model against the null model assuming that genetic differentiation is significantly different from zero is given; these are the delta Akaike information criterion (Δ_i_), the likelihood ratio (LR) and probability of error (P) for the hypothesis that SD_prov_ ≠ 0. Significant correlations (P < 0.05) are printed in bold, marginally significant correlations (P < 0.10) in italic bold letters*.

Significant genetic differentiations between provenances were found in three out of 22 ecophysiological traits only, namely the xylem pressure inducing 88% loss of hydraulic conductance (*P*_88_), vessel density (VD) and mean leaf size (*A*_leaf_; Table 3). The *P*_88_ values ranged from −3.60 MPa for the most vulnerable provenance from Germany (DE-BB) to −4.21 MPa for the most resistant provenance from Slovenia (SL), i.e., by 15% (Table S1). The differences in embolism resistance between provenances were, however, not mirrored in xylem anatomical adjustments. VD was significantly higher in the branch xylem of the most-productive provenances from Slovakia (SK) and the Ukraine (UA) while the largest leaves were found for the provenance adapted to the local climate of Schleswig Holstein (DE-SH; Table S1). Other provenances from Sweden (SE) and Czech Republic (CZ) produced on average 30% smaller leaves, which was not mirrored in adjustments of specific leaf area (SLA) or the Huber value (*A*_*S*_ : *A*_*L*_). In general, the leaf chemistry was uniform across the provenances.

Significant genetic differentiation between provenances (Table 3) was mirrored in high values of inter-population variance component (VC_inter_) for these three ecophysiological traits (*P*_88_, VD, *A*_leaf_) ranging from 8.38 to 14.02% (Table 3). Interestingly, all three measures of embolism resistance showed a higher variance between blocks (VC_block_ : ~25%) than between populations (VC_inter_ : ~10%). The remaining measured hydraulic traits, empirical specific conductivity ($K_{S}^{\text{emp}}$) and empirical leaf-specific conductivity ($K_{\text{L}}^{\text{emp}}$), likewise differed by ~10% between blocks but showed no variance between populations. Contrary to these hydraulic traits, all wood anatomical and calculated theoretical specific conductivity ($K_{\text{S}}^{\text{theo}}$) revealed no variance between blocks. For all traits measured excluding the growth-related variables, variability within provenances (CV_intra_) was about two times higher than variability between provenances (CV_inter_; Table 3).

### Trait-relatedness to climate at origin

**Figure 1:**
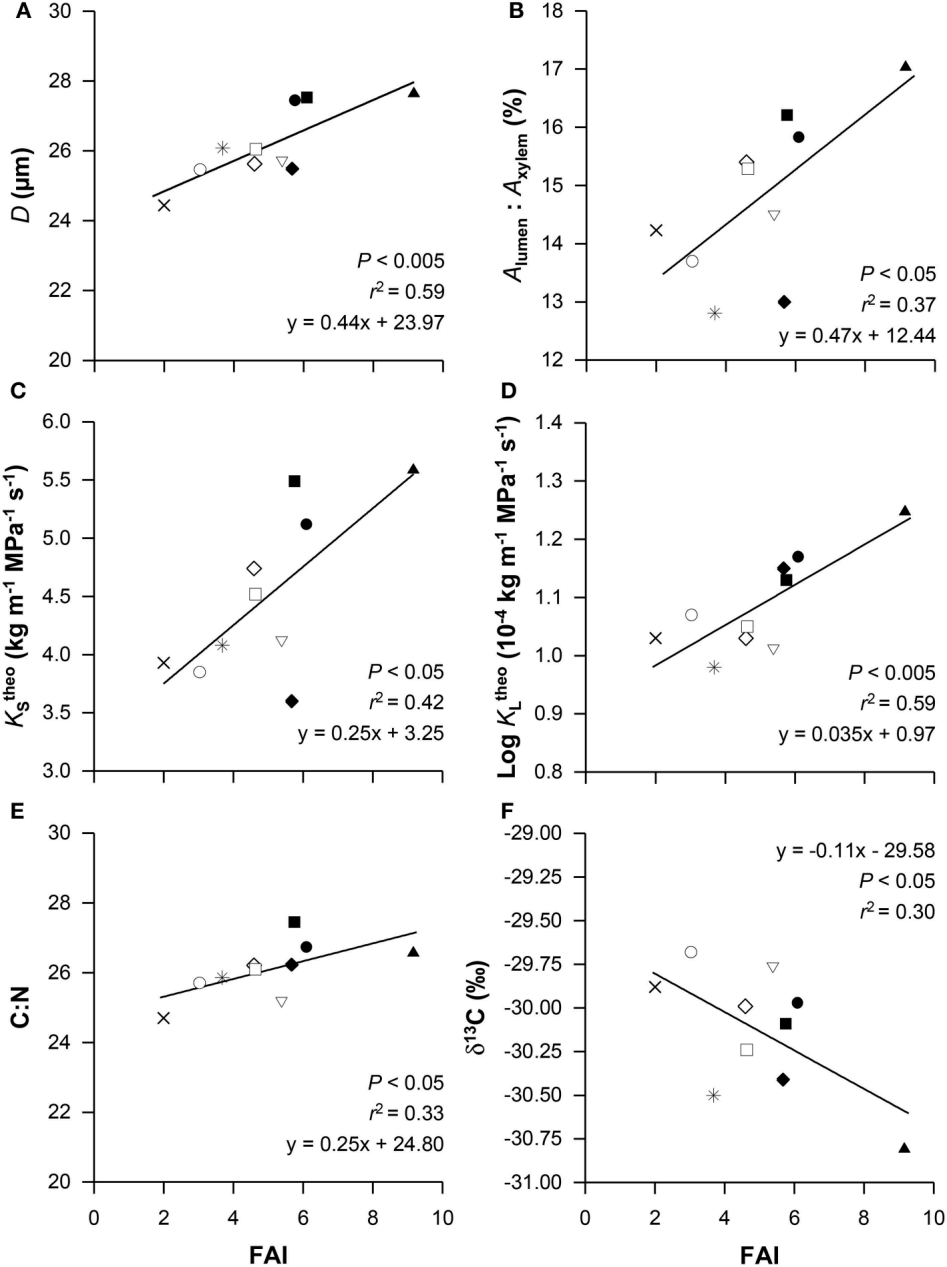
**Relationship between the forest aridity index (FAI) at the place of origin and vessel diameter (*D*, A), lumen to sapwood area ratio (*A*_*lumen*_ : *A*_*xylem*_, B), theoretical specific conductivity ($K_{\text{S}}^{\text{theo}}$, C), theoretical leaf-specific conductivity ($K_{\text{L}}^{\text{theo}}$, D), the carbon to nitrogen ratio (C:N, E) and the carbon isotope signature (δ^13^C, F)**. Given values are means per provenance. Each symbol represents one provenance, for provenance abbreviations see Table 1 (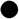
BG; 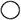
CZ; 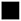
DE-BB; 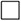
DE-SH; 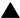
ES; 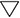
RO; 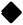
SE; 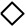
SK; 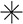
SL; 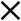
UA).

We observed several significant linear relationships between functional traits and the forest aridity index (FAI) as a measure of the climatic conditions at the place of provenance origin (Figure 1). These simple linear regression analyses were supported by linear mixed effect models confirming that FAI had a significant influence on seven of the 27 measured functional traits (Table 4). With increasing FAI, vessel diameter (*D*), lumen to sapwood area ratio (*A*_lumen_ : *A*_xylem_), theoretical specific conductivity ($K_{\text{S}}^{\text{theo}}$), theoretical leaf-specific conductivity ($K_{\text{L}}^{\text{theo}}$) and the C:N ratio significantly increased while the carbon isotope signature (δ^13^C) declined (Figures 1A–F; Table 4). Surprisingly, the three traits showing genetic differentiation between provenances (*P*_88_, VD and *A*_leaf_) were not significantly affected by FAI. However, our results show that provenances originating from dry habitats with high FAI values (e.g., BG, ES, DE-BB) form particularly wide vessels and have high lumen to sapwood area ratios (Figures 1A,B) resulting in a high $K_{\text{S}}^{\text{theo}}$ (Figure 1C) and $K_{\text{L}}^{\text{theo}}$ (Figure 1D) compared to the other provenances when grown at this humid site with high precipitation rates received during the entire growing season. The C:N ratio was also significantly positively related to FAI, while a strong significant negative relationship between FAI and δ^13^C was observed (Figure 1F).

**Table 4:** **Results of a linear mixed effect model examining the influence of the forest aridity index (FAI) at the place of origin on 27 measured parameters 19 years after planting of the provenance trial in Northern Germany**.

RELATEDNESS TO CLIMATE AT PROVENANCE ORIGIN									
Variable	*n*	α ± SE	β ± SE	*SD*_prov_	*SD*_block_	*SD*_resid_	Δ_i_	LR	*P*
**STAND CHARACTERISTICS**									
DBH	100	12.67 ± 1.71	−0.18 ± 0.32	1.84	0.21	1.30	1.59	0.41	0.52
Height	100	10.13 ± 1.16	0.001 ± 0.22	1.20	0.54	0.99	2.00	0.00	0.97
AGB	100	56.77 ± 16.19	−2.01 ± 3.04	17.45	0.00	11.91	1.47	0.53	0.47
**GROWTH-RELATED TRAITS**									
BAI	100	25.19 ± 7.78	−1.81 ± 1.46	7.95	0.82	9.92	0.25	1.75	0.19
ABI	100	2.99 ± 0.85	−0.11 ± 0.16	0.92	0.00	0.63	1.47	0.53	0.47
**HYDRAULIC PROPERTIES**									
*P*_12_	93	−2.15 ± 0.16	0.01 ± 0.03	0.11	0.17	0.28	1.93	0.07	0.80
*P*_50_	93	−3.13 ± 0.12	0.03 ± 0.02	0.08	0.14	0.22	0.43	1.57	0.21
*P*_88_	93	−4.06 ± 0.16	0.04 ± 0.03	0.14	0.07	0.29	0.20	1.80	0.18
$K_{\text{S}}^{\text{emp}}$	96	3.70 ± 0.37	0.004 ± 0.07	0.00	0.35	1.03	1.99	0.01	0.91
$K_{\text{S}}^{\text{theo}}$	98	3.24 ± 0.51	0.25 ± 0.09	0.10	0.00	1.69	4.26	6.26	**0.01**
$K_{\text{L}}^{\text{emp}*}$	95	2.04 ± 0.18	0.03 ± 0.04	0.00	0.16	0.51	0.98	1.02	0.31
$K_{\text{L}}^{\text{theo}*}$	95	2.09 ± 0.18	0.08 ± 0.03	0.00	0.00	0.61	4.03	6.03	**0.01**
**WOOD ANATOMY**									
BA	100	2.56 ± 0.15	−0.06 ± 0.03	0.00	0.00	0.51	3.17	5.17	**0.02**
*A*_growth_	98	16.42 ± 1.20	0.19 ± 0.22	0.00	0.00	4.07	1.29	0.71	0.40
*A*_lumen_ : *A*_xylem_	98	12.40 ± 1.03	0.48 ± 0.19	0.53	0.00	3.09	3.72	5.72	**0.02**
VD	98	245.92 ± 15.88	−0.27 ± 2.97	14.11	0.00	31.90	1.99	0.01	0.92
*D*	98	23.99 ± 0.67	0.43 ± 0.13	0.00	0.00	2.28	7.45	9.45	**0.002**
*D*_h_	98	32.05 ± 1.07	0.33 ± 0.20	0.58	0.00	3.18	0.84	2.84	***0.09***
**LEAF MORPHOLOGY**									
*A*_leaf_	100	23.98 ± 2.08	−0.20 ± 0.39	1.82	0.00	4.44	2.00	0.00	0.95
SLA	99	158.61 ± 12.16	−0.72 ± 2.28	0.00	8.79	39.16	1.89	0.11	0.74
*A*_S_ : $A_{\text{L}}^{*}$	100	0.74 ± 0.12	0.04 ± 0.02	0.03	0.03	0.42	1.04	3.04	***0.08***
**LEAF CHEMISTRY**									
C:N	100	24.80 ± 0.59	0.25 ± 0.11	0.35	0.00	1.73	3.12	5.12	**0.02**
δ^13^C	100	−29.59 ± 0.27	−0.11 ± 0.05	0.12	0.00	0.86	2.63	4.63	**0.03**
Ca_mass_	100	8.52 ± 0.53	−0.06 ± 0.10	0.00	0.15	1.79	1.66	0.34	0.56
K_mass_	100	4.54 ± 0.51	0.13 ± 0.10	0.39	0.00	1.30	0.09	2.09	0.15
Mg_mass_	100	1.62 ± 0.12	−0.03 ± 0.02	0.03	0.00	0.39	0.03	2.03	0.15
P_mass_	100	1.21 ± 0.05	0.001 ± 0.01	0.00	0.00	0.18	1.98	0.02	0.89

*Given are intercept α and slope β indicating the direction of the relationship for FAI with their corresponding standard errors (SE), standard deviation between provenances (SD_prov_) as an expression of the variability between provenances, standard deviation of block from the provenance mean showing the design variability, and the remaining unexplained variance of the residuals (SD_resid_). Further, the results from a likelihood ratio test of the LME against the null model are given; these are the delta Akaike information criterion (Δ_i_), the likelihood ratio (LR) and probability of error (P) for the hypothesis that FAI ≠ 0. Asterisks indicate traits that have been log transformed, for abbreviations see Table 2. Significant correlations of FAI on any given trait (P < 0.05) are printed in bold, marginally significant correlations (P < 0.10) in italic bold letters*.

### Determinants of aboveground growth performance

**Figure 2:**
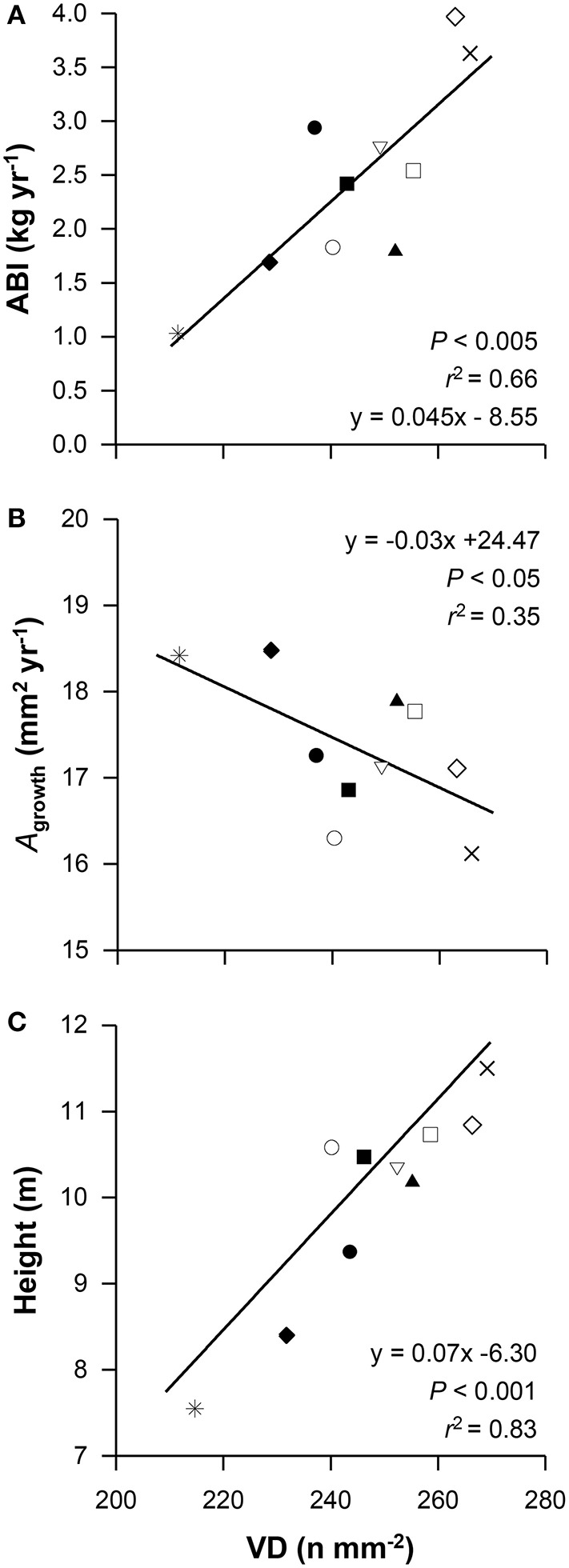
**Relationship between vessel density (VD) and aboveground biomass increment (ABI, A), annual branch sapwood area increment (*A*_*growth*_, B), and tree height (C)**. Given values are means per provenance, for symbol definition see Figure 1.

Among the xylem anatomical traits, only VD was positively related to aboveground biomass increment (ABI; *r*^2^ = 0.66, *P* < 0.005; Figure 2A) and negatively to branch growth rate (*A*_growth_; *r*^2^ = 0.35, *P* < 0.05; Figure 2B), which is a simplified measure of the annually produced branch sapwood, at the provenance level (*n* = 10). At the tree level (*n* = 100), only the negative relation between VD and *A*_growth_ could be confirmed (*r*^2^ = 0.26, *P* < 0.001; Table 5). Moreover, VD strongly increased with increasing tree height (*r*^2^ = 0.83, *P* < 0.001; Figure 2C). The remaining branch wood anatomical and hydraulic properties, however, varied independently from aboveground growth performance, and no correlations were found between ABI, embolism resistance and hydraulic efficiency, respectively (Table 5). However, a weak though significant negative relation was found for basal area increment (BAI) and *P*_50_ at the tree level (*r*^2^ = 0.08, *P* < 0.01; Table 5). Although we only observed weak or no relations between ABI and the wood anatomical and hydraulic traits, several of them were related to *A*_growth_ (Table 5). Hence, the expected trade-off between hydraulic efficiency and growth could be confirmed at branch level, but not at the tree level.

**Table 5:** **Pearson correlation coefficients for linear relationships between 27 functional trait variables at the tree level (*n* = 100)**.

	DBH	Height	AGB	BAI	ABI	*P*_12_	*P*_50_	*P*_88_	$K_{\text{S}}^{\text{emp}}$	$K_{\text{S}}^{\text{theo}}$	$K_{\text{L}}^{\text{emp}}$	$K_{\text{L}}^{\text{theo}}$	BA	*A*_*growth*_	*A*_*lumen*_ : *A*_*xylem*_	VD	*D*	*D*_*h*_	*A*_*S*_ : *A*_*L*_	*A*_*leaf*_	SLA	C:N	δ^13^C	Ca_*mass*_	K_*mass*_	Mg_*mass*_
Height	**0.56**																									
AGB	**0.98**	**0.66**																								
BAI	**0.73**	**0.31**	**0.74**																							
ABI	**0.98**	**0.66**	**1.00**	**0.74**																						
*P*_12_	ns	ns	ns	−**0.30**	ns																					
*P*_50_	ns	ns	ns	−**0.28**	ns	**0.82**																				
*P*_88_	ns	ns	ns	ns	ns	**0.37**	**0.79**																			
$K_{\text{S}}^{\text{emp}}$	ns	ns	ns	ns	ns	ns	ns	ns																		
$K_{\text{S}}^{\text{theo}}$	ns	ns	ns	ns	ns	ns	ns	ns	**0.44**																	
$K_{\text{L}}^{\text{emp}}$	ns	ns	ns	ns	ns	ns	ns	**0.28**	**0.57**	**0.39**																
$K_{\text{L}}^{\text{theo}}$	ns	ns	ns	ns	ns	ns	ns	ns	**0.37**	**0.73**	**0.77**															
BA	ns	ns	ns	ns	ns	ns	ns	ns	ns	ns	ns	ns														
*A*_growth_	ns	ns	ns	ns	ns	ns	ns	ns	***0.24***	***0.21***	**0.38**	**0.34**	ns													
*A*_lumen_ : *A*_xylem_	ns	ns	ns	ns	ns	ns	ns	ns	**0.36**	**0.94**	**0.30**	ns	ns	ns												
VD	ns	**0.36**	ns	ns	ns	ns	ns	ns	ns	ns	ns	ns	ns	**−0.51**	**0.49**											
*D*	ns	ns	ns	ns	ns	ns	ns	ns	**0.47**	**0.92**	**0.44**	**0.72**	ns	**0.39**	**0.79**	ns										
*D*_h_	ns	ns	ns	ns	ns	ns	ns	ns	**0.50**	**0.88**	**0.35**	**0.63**	ns	**0.34**	**0.71**	ns	**0.92**									
*A*_S_ : *A*_L_	ns	ns	ns	ns	ns	ns	ns	ns	ns	ns	**0.82**	**0.63**	−**0.30**	ns	ns	ns	**0.26**	ns								
*A*_leaf_	ns	ns	ns	ns	ns	ns	ns	ns	ns	ns	ns	**0.74**	ns	**0.36**	ns	ns	ns	ns	−**0.29**							
SLA	ns	ns	ns	ns	ns	**0.27**	ns	ns	ns	ns	−**0.31**	−**0.32**	**0.34**	*−**0.23***	ns	ns	ns	ns	**−0.46**	ns						
C:N	ns	ns	ns	ns	ns	ns	ns	ns	ns	ns	ns	ns	ns	***0.22***	ns	ns	ns	ns	ns	ns	ns					
δ^13^C	**0.26**	**0.26**	**0.28**	**0.29**	**0.28**	ns	ns	ns	ns	ns	ns	ns	ns	ns	ns	ns	ns	ns	ns	ns	**−0.34**	−**0.32**				
Ca_mass_	ns	ns	ns	ns	ns	ns	ns	ns	ns	ns	ns	ns	ns	ns	ns	ns	ns	ns	ns	ns	ns	ns	ns			
K_mass_	ns	ns	ns	ns	ns	ns	ns	ns	ns	ns	ns	ns	ns	ns	ns	ns	ns	ns	ns	ns	**0.41**	**0.41**	ns	ns		
Mg_mass_	ns	ns	ns	ns	ns	ns	ns	ns	ns	ns	ns	ns	ns	ns	ns	ns	ns	ns	ns	ns	**0.37**	ns	ns	ns	ns	
P_mass_	ns	ns	ns	ns	ns	ns	ns	ns	ns	ns	ns	ns	ns	ns	ns	ns	ns	ns	ns	ns	ns	**0.40**	ns	ns	**0.51**	ns

*Significance levels: ns: non−significant; bold italic letters: P < 0.05; bold letters: P < 0.01; bold underlined letters: P < 0.001. See Table 1 for definition of abbreviations*.

### Inter-relationships between functional traits

**Figure 3:**
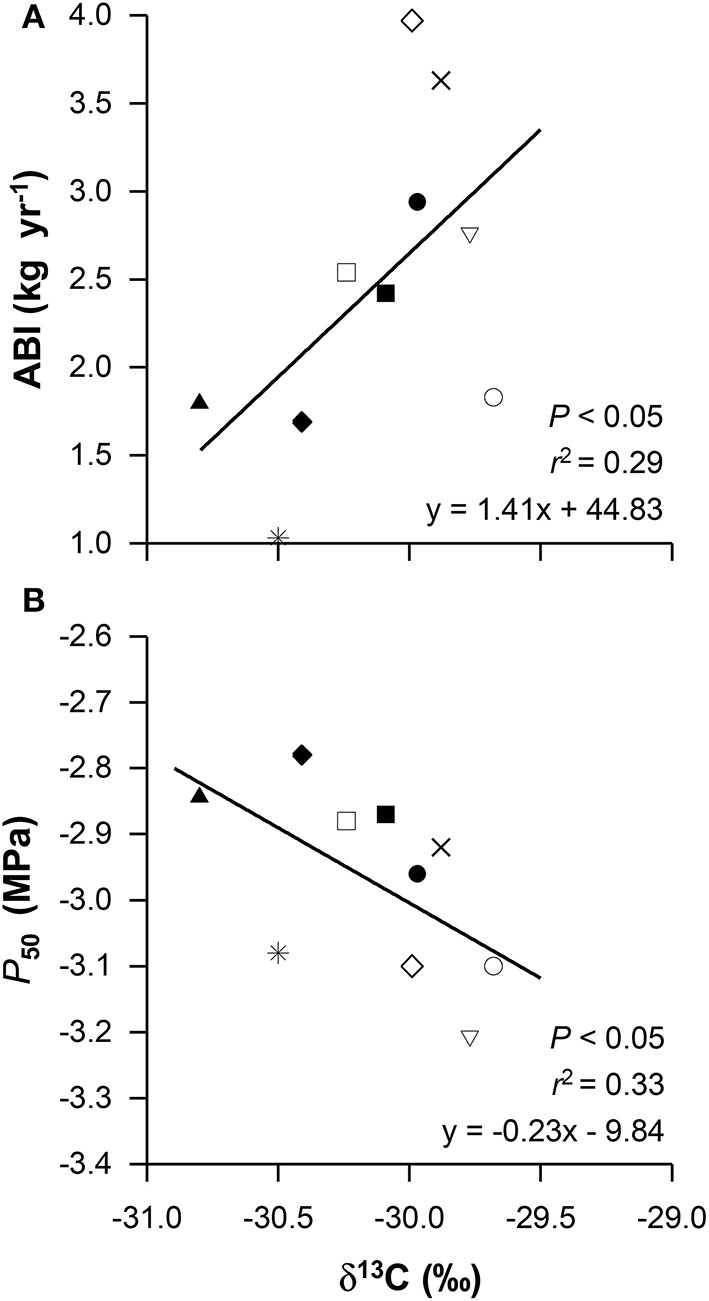
**Aboveground biomass increment (ABI, A) and the xylem pressure causing 50% loss of hydraulic conductivity (*P*_50_, B) in relation to the carbon isotope signature**. Given values are means per provenance, for symbol definition see Figure 1.

**Figure 4:**
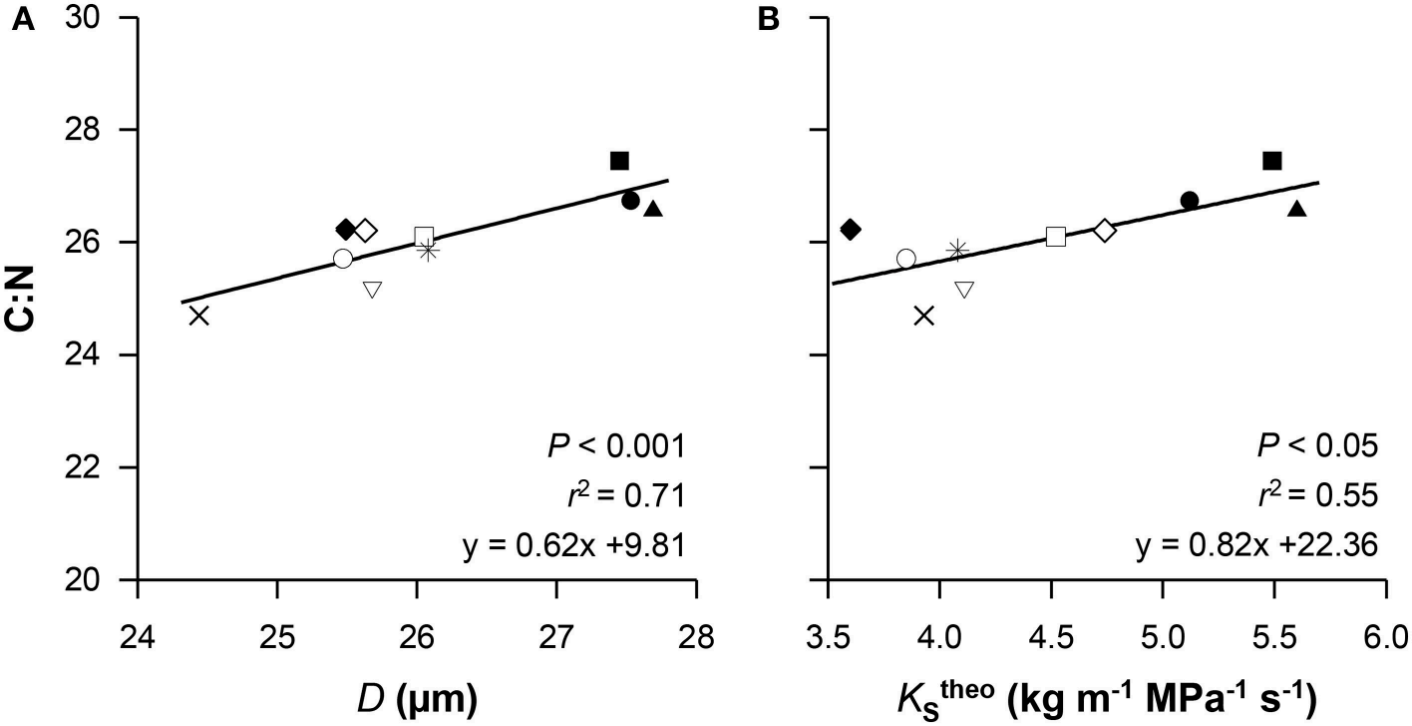
**The carbon to nitrogen ratio (C:N) in relation to vessel diameter (*D*, A) and theoretical specific conductivity ($K_{\text{S}}^{\text{theo}}$, B)**. Given values are means per provenance, for symbol definition see Figure 1.

We found that hydraulic efficiency in beech depends on the xylem properties. The vessel lumen to sapwood area ratio (*A*_lumen_ : *A*_xylem_), annual branch growth rate (*A*_growth_), vessel diameter (*D*) and hydraulically-weighed vessel diameter (*D*_h_) were closely positively related to empirical specific conductivity ($K_{\text{S}}^{\text{emp}}$) and empirical leaf-specific conductivity ($K_{\text{L}}^{\text{emp}}$). In contrast, correlations between vessel density (VD) and the hydraulic traits $K_{\text{S}}^{\text{emp}}$ or $K_{\text{S}}^{\text{theo}}$ were not found (Table 5). Furthermore, none of the wood anatomical traits were related to embolism resistance and the expected trade-off between hydraulic conductivity and embolism resistance was absent. In addition, we found significant links between functional leaf traits such as specific leaf area (SLA) and empirical as well as theoretical leaf-specific conductivity ($K_{\text{L}}^{\text{emp}}$, $K_{\text{L}}^{\text{theo}}$). The carbon isotope signature (δ^13^C) was positively correlated with ABI (fast growth was associated with a frequent stomatal closure) but negatively with the *P*_50_ value (Figures 3A,B). The provenances with the highest growth rates (UA, SK, and BG) exhibited highest carbon isotope signature (*r*^2^ = 0.29, *P* < 0.05; Figure 3A). The foliar C:N ratio was positively related to vessel diameter (*D*) and the theoretical specific conductivity ($K_{\text{S}}^{\text{theo}}$; Figures 4A,B), indicating that higher growth rates (associated with a high C:N ratio) were indirectly related to xylem hydraulic properties of the branch wood.

## Discussion

### Genetic differentiation

Only recently, a growing number of studies have investigated the intraspecific genetic differentiation in ecophysiological traits between populations (e.g., Bresson et al., 2011; Lamy et al., 2011; Eilmann et al., 2014; Aranda et al., 2015; Schreiber et al., 2015). Despite this positive trend, common-garden studies that have reported anatomical or hydraulic traits are still scarce (Anderegg and Meinzer, 2015). Our study on ten European beech provenances native to different localities in Europe revealed little intraspecific variation of the ecophysiological traits covered. In agreement with former common-garden experiments we observed no significant genetic differences between populations for the xylem pressure causing 50% loss of conductivity. The same pattern has been described for conifers (Sáenz-Romero et al., 2013; Lamy et al., 2014) as well as for beech (Wortemann et al., 2011, but see Aranda et al., 2015). Contrary to these studies, however, we observed significant genetic differentiation for the xylem pressure inducing 88% loss of hydraulic conductance (*P*_88_), which corresponds to the threshold for catastrophic hydraulic failure leading to irreversible drought-induced dysfunction in angiosperms (Urli et al., 2013; Li et al., 2015). In our study, genetic differentiation was further found for the xylem anatomical trait vessel density and the foliar trait mean leaf size. Our first hypothesis postulating that wood hydraulic properties are under genetic control due to local adaptation is further supported by the observation that most wood anatomical and derived hydraulic traits as well as some foliar traits were closely related to the climate at provenance origin. These results may indicate that wood anatomical and hydraulic traits of beech reflect genetic differentiation between provenances although no significant intraspecific differences became evident. This is in line with the observation that several wood properties related to vessel size are predominantly under genetic predisposition (Eilmann et al., 2014).

However, the high intra-population variability in all ecophysiological traits covered, which was on average two to three times higher than inter-population variability, is also a strong evidence for the high genetic diversity within provenances. For example, Aranda et al. (2015) stated that beech exhibits a high degree of intra-population genetic variability for embolism resistance. This indicates that European beech might have a high potential capacity to adapt to climate change. In agreement hereon, both hydraulic efficiency and embolism resistance were unrelated to climatic aridity at the place of provenance origin and showed the highest variance within a given provenance (~65%), followed by the second highest variance between replicated blocks (~25%) due to micro-environmental effects including within-canopy variability, and only a comparatively low variance between populations (~10%). This likewise confirms that beech exhibits a high degree of intra-population genetic variability for embolism resistance. Surprisingly, provenances originating from drought prone habitats (e.g., ES, BG, and DE-BB) grown at our site do not represent the most drought tolerant provenances in terms of functional trait adaptation as expected (see Eilmann et al., 2014). This finding is in agreement with former studies quantifying the high phenotypic plasticity of embolism resistance across environmental gradients (Herbette et al., 2010; Wortemann et al., 2011; Schuldt et al., 2016). Moreover, the vessel density of the branch xylem seems to play an outstanding role in the adaptation of the branch hydraulic system to different climates. Likewise with embolism resistance, vessel density varied independently of climatic conditions at the place of origin but was significantly different among provenances presumably as a consequence of differences in branch height. European beech trees may accordingly hold the capacity to adapt to local climate conditions by modifying their branch hydraulic traits primarily through adjustments in the relative abundance of vessels. This assumption is further supported by the close relation between vessel density and productivity as tree growth performance is hypothesized to scale with hydraulic efficiency (Tyree, 2003), which contrary to our results has been confirmed for both temperate and tropical tree species (Hajek et al., 2014; Hoeber et al., 2014; Kotowska et al., 2015).

### Functional trade-offs

It has been suggested that embolism resistance decreases with growth rate due to conflicting carbon allocation either to the construction of thicker cell walls, or to the building of foliar and axial tissues destined to increase canopy carbon gain (Cochard et al., 2007). However, empirical data from different species or genotypes do not unequivocally support this trade-off. For example, Fichot et al. (2010) reported on embolism-resistant genotypes of poplar which grew faster than more vulnerable genotypes. We observed no relation between *P*_50_ in branches and aboveground biomass increment across the provenances contradicting the hypothesized relation between embolism resistance and growth, which should trade-off with hydraulic efficiency. Several recent studies also failed to detect this relationship between embolism resistance and growth rate (Sterck et al., 2012; Hajek et al., 2014; Guet et al., 2015), indicating that embolism resistance is partly decoupled from hydraulic efficiency and biomass production (Fichot et al., 2015). In agreement hereon and in line with our results, hydraulic safety seems mostly decoupled from hydraulic efficiency, both at the inter-specific (Gleason et al., 2016) and intra-specific level (Schuldt et al., 2016; but see Hajek et al., 2014). In addition to these anticipated trade-offs it is necessary to include the foliage functionality for a holistic understanding of growth-related processes (Carlquist, 2012). In our sample, the *P*_50_ and *P*_88_ values decreased in parallel with increasing carbon isotope signature. The most embolism resistant provenances thus presumably had to close their stomata more frequently at the expense of significantly reduced carbon assimilation. This observation is contradicting observations of the functional coordination between vulnerability to xylem cavitation and the regulation of stomatal conductance (Sparks and Black, 1999; Brodribb et al., 2002). Either a sensitive stomatal regulation or simply a lower water demand as a consequence of smaller stature of drought prone provenances may explain this trait interrelation.

Moreover, the *P*_50_ and *P*_88_ values were not related to xylem anatomical traits like vessel diameter or vessel density as evidenced in several studies among and across species (Domec et al., 2010; Hajek et al., 2014). In contrast, the range of variation was small in our study and might have hampered a significant relationship as likewise described by other authors who also failed to detect a relation between vessel diameter and embolism resistance in closely related genotypes or different hybrids of poplar (Cochard et al., 2007; Fichot et al., 2010). These findings support the growing evidence that variation in *P*_50_ is mainly determined by other wood structural properties such as the topology of the xylem network (Loepfe et al., 2007; Martínez-Vilalta et al., 2012) as well as the pit membrane morphology (Plavcova et al., 2013; Li et al., 2016). The relation with vessel size in studies covering a sufficiently steep range in diameters is thus rather indirect as already speculated by Tyree and Sperry (1989).

In our study, provenances originating from drought-prone habitats (e.g., Spain) were most vulnerable to xylem cavitation. This contradicts the general observation of a less vulnerable structure for provenances originating from drought-prone sites (Rose et al., 2009; Robson et al., 2012). Surprisingly, the Spanish provenance developed the largest vessels of all ten provenances under these favorable environmental conditions contrary to our expectation. Due to the genetic control of this trait, beech provenances adapted to drier climates seem to over-develop their vascular system when grown in more humid environments. However, this did not translate into a better growth performance of these provenances.

## Conclusion

Our study on ten beech provenances from all over Europe revealed that most wood anatomical and derived hydraulic traits and some foliar traits are under genetic predisposition according to significant differentiation between provenances or the relation with climatic aridity at the place of provenance origin. Although a certain degree of genetic differentiation was observed for embolism resistance, the high ratio of intra- vs. inter-population variance suggests that the genotypic variability of this trait is high between genotypes of a given provenance. In European beech, this high adaptive capacity in xylem function seems predominantly to be a consequence of adjusting vessel number but not necessarily vessel size. Nevertheless, we could not confirm the anticipated trade-off between hydraulic efficiency, xylem safety and growth in agreement with several recent studies. It further remains unclear if the observed high adaptive capacity of young European beech tree individuals can be extrapolated to old-growth forest trees, potentially enabling them to withstand increased drought by a flexible hydraulic response.

## Author contributions

BS and GvW designed the study, PH, DK, and BS collected the field samples, DK and PH performed the hydraulic, wood anatomical and leaf morphological measurements and PH, BS, and SD analyzed the data and performed the statistical analyses. PH and BS wrote the first version of the manuscript, which was intensively discussed and revised by all authors.

### Conflict of interest statement

The authors declare that the research was conducted in the absence of any commercial or financial relationships that could be construed as a potential conflict of interest.
