# Supplementary material for: Intraspecific Variation in Wood Anatomical, Hydraulic, and Foliar Traits in Ten European Beech Provenances Differing in Growth Yield
Source: Front Plant Sci. 2016 Jun 15;7:791. doi: 10.3389/fpls.2016.00791 (PMC4909056; doi:10.3389/fpls.2016.00791)
Supplement: Supplementary file 1 [file DataSheet1.pdf]

Intraspecific variation in wood anatomical, hydraulic and foliar traits in ten European beech provenances differing in growth yield

Peter Hajek, Daniel Kurjak, Georg von Wühlisch, Sylvain Delzon and Bernhard Schuldt

Supplementary material

Table S1: Stand characteristics, growth-related, hydraulic, wood anatomical and leaf morphological traits as well as leaf chemistry of the ten investigated provenances measured 19 years after planting. One-way ANOVA was used to quantify the genetic differentiation of the investigated traits. The General Linear Hypotheses (glht) procedure with Tukey's post hoc test was applied to detect significant differences in the analysed trait means among the ten provenances. Different letters indicate significant differences between provenances ($P < 0.05$, means ± SE). For abbreviations see Table 2.

	Country																			
Variable	BG		CZ		ES		DE-BB		DE-SH		RO		SE		SK		SL		UA	
**Stand characteristics**																				
DBH	12.82 ± 0.32	ab	10.65 ± 0.29	cd	10.30 ± 0.42	de	11.78 ± 0.23	bd	11.92 ± 0.35	bd	12.55 ± 0.40	bcf	10.55 ± 0.43	d	14.63 ± 0.64	a	8.51 ± 0.51	e	13.83 ± 0.40	af
Height	10.70 ± 0.37	ab	9.49 ± 0.44	bc	10.32 ± 0.30	ab	10.59 ± 0.37	ab	10.85 ± 0.30	ab	10.45 ± 0.46	ab	8.52 ± 0.27	cd	10.96 ± 0.40	ab	7.67 ± 0.23	d	11.62 ± 0.20	a
AGB	55.87 ± 3.95	ab	34.69 ± 2.39	cd	34.34 ± 3.08	cd	45.90 ± 2.07	bd	48.27 ± 3.58	bd	52.20 ± 3.59	ab	32.20 ± 3.14	cd	75.41 ± 6.74	e	19.48 ± 2.68	c	69.03 ± 4.31	ae
**Growth-related traits**																				
BAI	14.47±2.41	ab	15.72±1.87	ab	4.34±0.95	a	19.38±2.11	bcc	13.05±3.33	ab	22.55±2.97	bd	9.60±1.85	ab	33.36±5.87	d	5.68±1.44	ac	23.20±4.99	bd
ABI	2.94 ± 0.21	ab	1.82 ± 0.13	cd	1.81 ± 0.16	cd	2.42 ± 0.11	bd	2.54 ± 0.19	bd	2.75 ± 0.19	ab	1.69 ± 0.17	cd	3.97 ± 0.35	e	1.02 ± 0.14	c	3.63 ± 0.23	ae
**Hydraulic properties**																				
P_{12}	-1.94 ± 0.12	a	-2.28 ± 0.14	a	-2.11 ± 0.12	a	-2.07 ± 0.07	a	-1.91 ± 0.04	a	-2.38 ± 0.05	a	-1.94 ± 0.12	a	-2.30 ± 0.11	a	-2.09 ± 0.11	a	-2.01 ± 0.11	a
P_{50}	-2.96 ± 0.11	ab	-3.1 ± 0.09	ab	-2.84 ± 0.10	ab	-2.87 ± 0.05	ab	-2.88 ± 0.03	ab	-3.21 ± 0.06	a	-2.78 ± 0.11	b	-3.10 ± 0.06	ab	-3.08 ± 0.09	ab	-2.92 ± 0.11	ab
P_{88}	-3.98 ± 0.14	ab	-3.92 ± 0.06	ab	-3.69 ± 0.14	b	-3.60 ± 0.05	b	-3.85 ± 0.05	ab	-4.03 ± 0.08	ab	-3.63 ± 0.11	b	-3.89 ± 0.06	ab	-4.21 ± 0.09	a	-3.84 ± 0.13	ab
K_S^{emp}	4.21 ± 0.43	a	3.69 ± 0.16	a	3.20 ± 0.29	a	4.21 ± 0.42	a	4.10 ± 0.21	a	3.47 ± 0.43	a	3.61 ± 0.30	a	3.36 ± 0.44	a	3.54 ± 0.36	a	3.56 ± 0.23	a
K_S^{theo}	5.12 ± 0.40	a	3.85 ± 0.44	a	5.60 ± 0.63	a	5.49 ± 0.61	a	4.52 ± 0.46	a	4.11 ± 0.48	a	3.60 ± 0.33	a	4.74 ± 0.90	a	4.08 ± 0.44	a	3.93 ± 0.44	a
K_L^{emp}	10.62 ± 2.22	a	10.34 ± 1.77	a	10.62 ± 1.50	a	11.24 ± 1.84	a	10.07 ± 1.17	a	8.34 ± 1.42	a	14.17 ± 2.42	a	9.46 ± 2.09	a	7.75 ± 1.09	a	9.28 ± 1.22	a
K_L^{theo}	16.46±3.28	a	19.45±4.65	a	15.1±4.98	a	19.73±2.99	a	11.77±2.05	a	15.73±2.39	a	12.2±1.51	a	16.36±5.91	a	10.68±2.01	a	11.96±2.03	a
**Wood anatomy**																				
BA	2.30±0.15	a	2.50±0.27	a	2.10±0.10	a	2.20±0.13	a	2.10±0.10	a	2.20±0.13	a	2.00±0.00	a	2.20±0.13	a	2.4±0.16	a	2.50±0.27	a
A_{growth}	17.26±1.09	a	16.30±2.01	a	17.91±0.91	a	16.86±1.10	a	17.77±1.15	a	17.11±1.44	a	18.48±0.92	a	17.11±1.51	a	18.42±1.61	a	16.12±1.43	a
$A_{lumen} : A_{xylem}$	15.83 ± 0.79	a	13.69 ± 0.80	a	17.06 ± 1.15	a	16.20 ± 0.93	a	15.29 ± 0.90	a	14.48 ± 0.97	a	13.00 ± 0.77	a	15.40 ± 1.62	a	12.81 ± 0.66	a	14.23 ± 0.88	a
VD	237.00 ± 7.78	ab	240.38 ± 10.99	ab	252.04 ± 9.79	ab	243.03 ± 5.44	ab	255.39 ± 12.57	ab	249.2 ± 9.63	ab	228.57 ± 9.41	ab	263.21 ± 14.80	b	211.49 ± 7.66	a	265.97 ± 10.93	b
D	27.53 ± 0.56	a	25.47 ± 0.59	a	27.69 ± 0.59	a	27.45 ± 0.84	a	26.05 ± 0.73	a	25.68 ± 0.72	a	25.49 ± 0.52	a	25.63 ± 1.27	a	26.08 ± 0.56	a	24.44 ± 0.54	a
D_h	35.02 ± 0.67	a	32.52 ± 1.01	a	34.89 ± 0.89	a	35.57 ± 1.18	a	33.72 ± 0.98	a	32.61 ± 0.85	a	32.42 ± 0.73	a	32.68 ± 1.61	a	34.75 ± 1.02	a	32.54 ± 0.88	a
**Leaf morphology**																				
A_{leaf}	23.69 ± 1.19	ab	21.87 ± 0.91	ab	23.64 ± 1.15	ab	23.85 ± 1.64	ab	27.65 ± 1.98	a	24.69 ± 1.25	ab	19.68 ± 1.13	b	25.82 ± 1.37	ab	24.91 ± 1.61	ab	23.01 ± 1.49	ab
SLA	144.36 ± 8.67	a	141.85 ± 10.48	a	151.17 ± 8.43	a	159.47 ± 5.42	a	153.3 ± 4.83	a	147.33 ± 12.46	a	161.08 ± 17.40	a	157.22 ± 16.75	a	181.81 ± 13.32	a	152.74 ± 19.95	a
A_S:A_L	3.02 ± 0.53	a	2.79 ± 0.44	a	3.19 ± 0.25	a	2.60 ± 0.27	a	2.44 ± 0.24	a	2.35 ± 0.21	a	3.77 ± 0.42	a	2.71 ± 0.38	a	2.27 ± 0.26	a	2.76 ± 0.49	a
**Leaf chemistry**																				
C:N	26.74 ± 0.57	ab	25.71 ± 0.62	ab	26.61 ± 0.53	ab	27.45 ± 0.53	b	26.10 ± 0.55	ab	25.15 ± 0.67	ab	26.23 ± 0.52	ab	26.21 ± 0.51	ab	25.86 ± 0.54	ab	24.70 ± 0.36	a
$\delta^{13}C$	-29.97 ± 0.37	a	-29.68 ± 0.32	a	-30.80 ± 0.26	a	-30.09 ± 0.22	a	-30.24 ± 0.32	a	-29.77 ± 0.35	a	-30.41 ± 0.15	a	-29.99 ± 0.16	a	-30.50 ± 0.25	a	-29.89 ± 0.23	a
Ca_{mass}	7.32 ± 0.43	a	8.72 ± 0.65	a	8.44 ± 0.65	a	7.74 ± 0.41	a	8.80 ± 0.52	a	8.14 ± 0.49	a	8.35 ± 0.64	a	8.50 ± 0.56	a	7.86 ± 0.66	a	8.47 ± 0.69	a
K_{mass}	6.53 ± 0.68	a	4.94 ± 0.30	ab	5.45 ± 0.20	ab	5.23 ± 0.27	ab	5.55 ± 0.46	ab	5.21 ± 0.47	ab	4.53 ± 0.35	b	5.30 ± 0.25	ab	4.47 ± 0.43	b	4.80 ± 0.47	ab
Mg_{mass}	1.36 ± 0.05	a	1.41 ± 0.08	a	1.23 ± 0.11	a	1.41 ± 0.07	a	1.52 ± 0.10	a	1.51 ± 0.21	a	1.70 ± 0.19	a	1.51 ± 0.08	a	1.63 ± 0.12	a	1.44 ± 0.14	a
P_{mass}	1.27 ± 0.07	a	1.26 ± 0.03	a	1.22 ± 0.03	a	1.27 ± 0.03	a	1.20 ± 0.02	a	1.13 ± 0.13	a	1.18 ± 0.04	a	1.24 ± 0.05	a	1.23 ± 0.05	a	1.18 ± 0.05	a

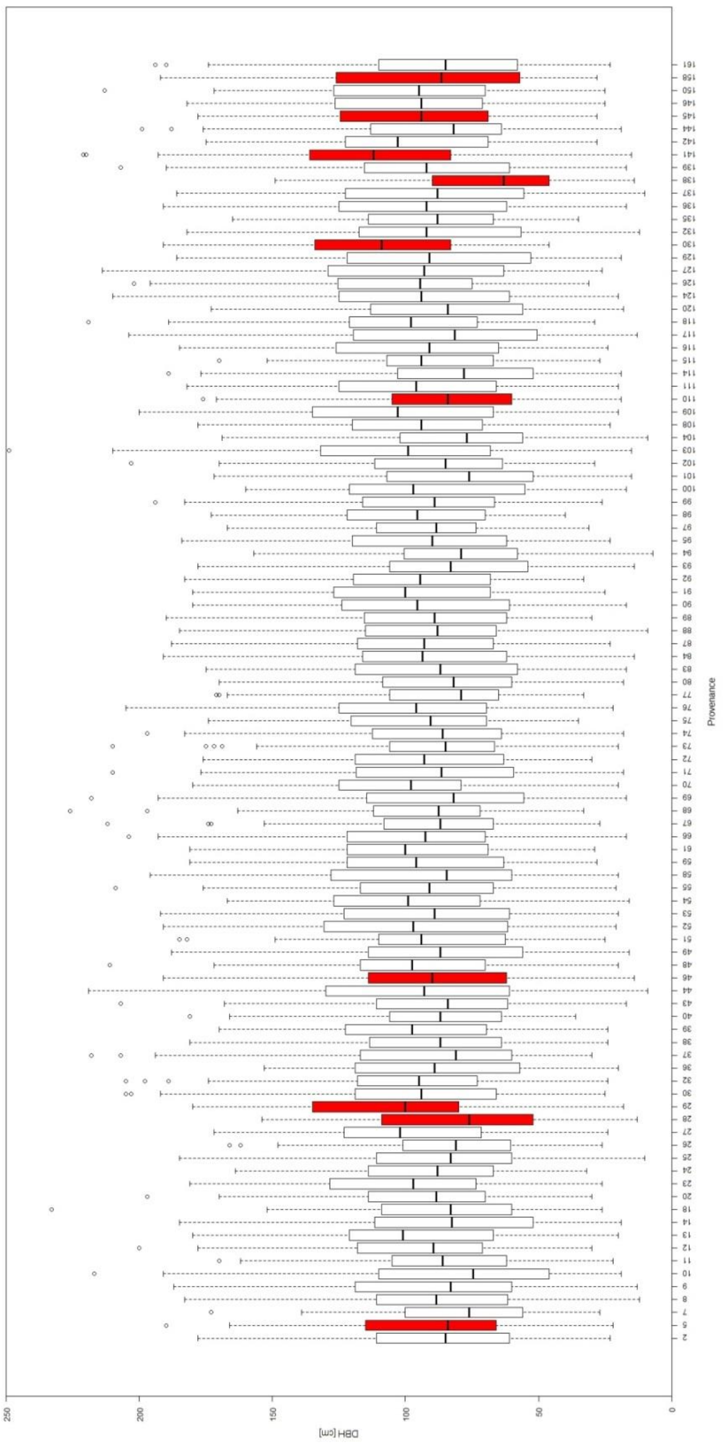

Figure S1: Overview of the range in diameter at breast height (DBH, cm) of all 100 available beech provenances planted in the common-garden experiment in Northern Germany. The ten selected provenances according to differences in size and thus growth rate are marked red.

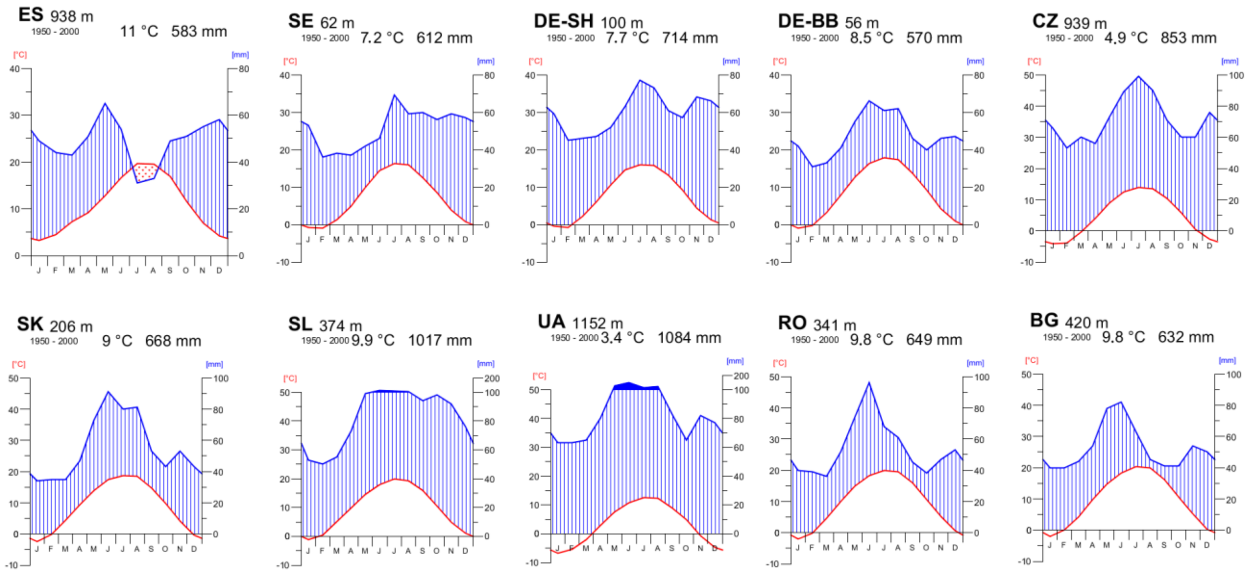

Figure S2: Climate diagrams at the place of origin of the ten selected beech provenances planted in a common-garden experiment in Northern Germany. Mean annual climate data from 1950 to 2000 were obtained from the WorldClim database with 30 arc-seconds resolution (Hijmans et al., 2005).
